# Relationship Between Electronegativity of the Extra-Framework Cations and Adsorption Capacity for CO_2_ Gas on Mordenite Framework

**DOI:** 10.1021/acs.inorgchem.4c04062

**Published:** 2024-12-26

**Authors:** Soojin Lee, Hyunseung Lee, Huijeong Hwang, Donghoon Seoung, Hyeonsu Kim, Pyosang Kim, Yongmoon Lee

**Affiliations:** † Department of Geological Sciences, Pusan National University, Busan 46241, Korea; ‡ School of Environment and Energy Engineering, Gwangju Institute of Science and Technology, Gwangju 61005, Korea; § Department of Earth and Environmental Sciences, Chonnam National University, Gwangju 61186, Korea; ∥ Institute for Future Earth Environment, Pusan National University, Busan 46241, Korea

## Abstract

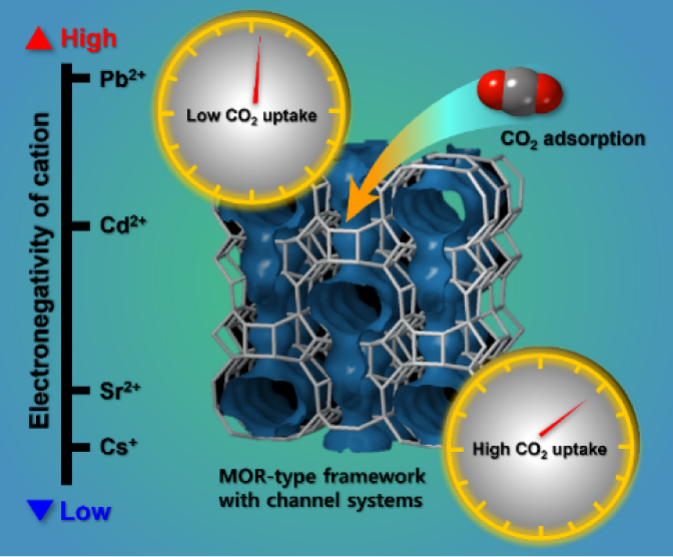

Synthetic mordenite is widely used as a molecular sieve, adsorbent, and catalyst. To enhance these functionalities, it is crucial to understand the ion-exchange properties and cation-exchange sites of the zeolite. In this study, we analyzed the structural changes in fully Cs-, Sr-, Cd-, and Pb-exchanged mordenite by using synchrotron X-ray powder diffraction under ambient conditions. Rietveld structure refinement revealed that the Cs^+^ cation is predominantly located near the 8-membered ring (8MR) due to its low electronegativity and hydration energy. In contrast, divalent cations such as Sr^2+^ and Cd^2+^ cations, with higher hydration energies compared to monovalent cations, are present as hydrated ions at the center of the 12-membered ring along the *c*-axis (12MRc). Pb^2+^ ions, due to their higher electronegativity than the framework atoms, exhibit a strong affinity for the electron cloud of framework oxygen atoms, which positions them close to the wall of the 12MRc. The observed differences in the locations of the extra-framework cations are attributed to electrostatic and hydration effects. Furthermore, the CO_2_ adsorption capacity was assessed based on the type and site of exchangeable cations. The findings indicate that an increase in the CO_2_ adsorption capacity correlates with the number of cations that can effectively interact with CO_2_.

## Abstract 2

Changes in the crystal structure of MORs result from the extra-framework cation properties, such as electronegativity and hydration energy, which affect the CO_2_ gas adsorption capacity. The CO_2_ uptake increases with decreasing electronegativity and increasing polarizing power of cations in the 8MRb. Therefore, our research showed that the physicochemical properties of cations are essential for the CO_2_ adsorption mechanism with the MOR framework.

## Introduction

Toxic heavy metals like cadmium (Cd) and lead (Pb) are not biodegradable.^1−3^ Similarly, radioactive isotopes like 137Cs and 90Sr have long half-lives of about 30 years and are highly soluble in water, increasing their potential for environmental and human body accumulation.^4,5^ Additionally, anthropogenic carbon dioxide (CO_2_) emissions significantly contribute to climate change, accounting for approximately 55% of global warming.^6,7^ The extensive impact of these pollutants underscores the urgent need for effective treatments to mitigate their release into the environment.

Heavy metal and radioactive cations can be removed from water through chemical and physical methods.^2,8^ Ion-exchange presents a viable alternative to these conventional techniques.^9,10^ In CO_2_ capture technologies, physisorption using porous materials stands out as a promising method, generally offering fast CO_2_ adsorption kinetics and the advantage of low energy regeneration costs.^11^ However, developing CO_2_ physisorbents that provide high CO_2_ adsorption capacity, high selectivity, and good reusability remains a significant challenge. Therefore, it is crucial to advance the development of solid porous adsorbents that achieve these properties to effectively combat environmental pollution.^12^

**Figure 1:**
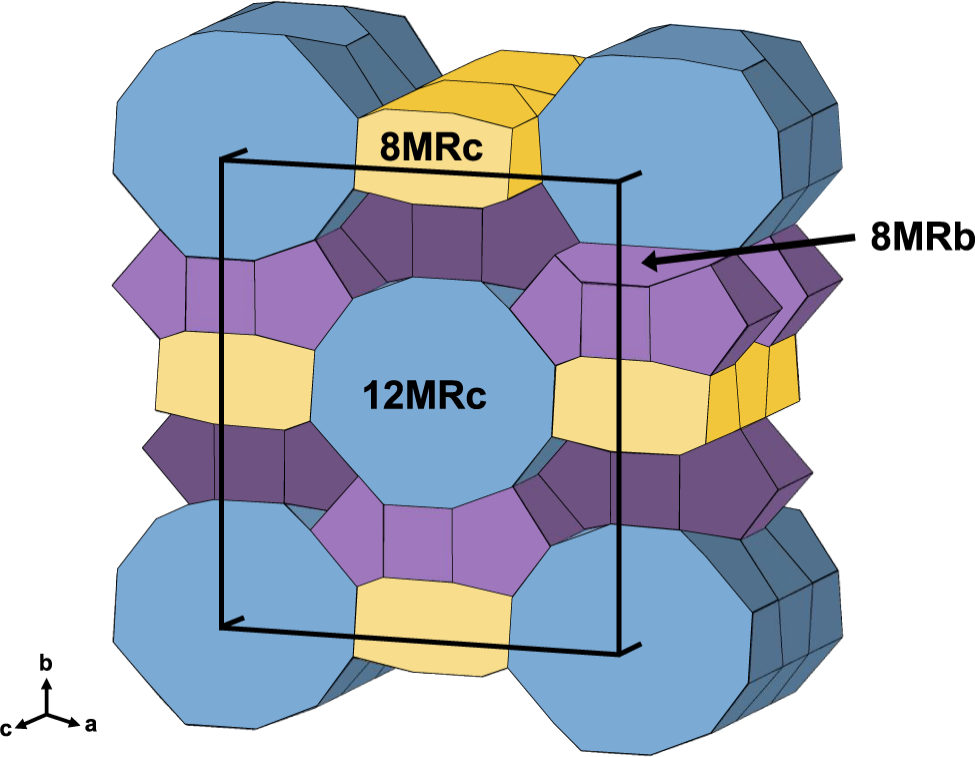
Structure of the mordenite 3D framework with unit cell outlines. Two channel systems are oriented along the *c*-axis: 12MRc and 8MRc, shown in blue and yellow, respectively. These two channels are interconnected by 8MRb parallel to the *b*-axis, represented in violet.

Mordenite is one of the siliceous zeolites (Si/Al > 5), with an ideal composition of Na_8_Al_8_Si_40_O_96_·24H_2_O. The structure of mordenite comprises edge-sharing 5-membered rings of tetrahedra, forming chains along the *c*-axis.^13^ Its framework is built by sheets of 6-membered rings of tetrahedra parallel to the (100) plane, linked by 4-membered rings of tetrahedra along the (100) direction. This arrangement creates two systems of channels along the (001) direction: a large 12-membered ring (12MRc) and a strongly compressed 8-membered ring (8MRc). These channels are interconnected along the (010) direction by side pockets accessible through 8-membered rings (8MRb) and staggered by c/2 at the intersection with the 8MRc (Figure 1).^14,15^ Extra-framework cations (EFCs) in mordenite primarily occupy three sites: (1) close to the 4MRc, (2) centering the 12MRc and 8MRc, and (3) centering the 8MRb at the intersection between the 12MRc channel and the side pocket along the (010) direction.^16,17^

The CO_2_ adsorption behavior in zeolites can be categorized into two groups based on the number of tetrahedra in ring.^7^ The first group consists of 10MR and 12MR zeolites, with pore sizes ranging from 4.5 to 6 Å and 6 to 8 Å, respectively, where adsorption is primarily influenced by electrostatic interactions.^7,18^ The second group comprises 8MR zeolites with pore sizes of between 3 and 4.5 Å. In this group, diffusion and size exclusion effects play additional roles due to the similarity in size to CO_2_ gas molecules (∼3.3 Å).^7,19,20^ For each type of zeolite, there is a complex balance between basicity, polarizing power, and steric effects determined by the nature of the extra-framework cations that influence the CO_2_ adsorption capacity.^7^ The adsorption behavior of zeolites can be modified through ion-exchange processes, which alter the nature and distribution of the cations. To understand the correlation between extra-framework cations and CO_2_ adsorption capacity, studies have integrated the Rietveld refinement method with CO_2_ adsorption isotherms. Through this combination, it is possible to gain detailed insights into the structural changes and the effects on the CO_2_ adsorption properties. Here, we report structures of fully Na^+^-, Cs^+^-, Sr^2+^-, Cd^2+^-, and Pb^2+^-exchanged mordenites and compare them to the CO_2_ gas adsorption capacities.

## Experimental Section

### Sample Preparation

A synthetic sample of Na-mordenite (Na-MOR, Na_6.6_Al_6.6_Si_41.4_O_96_·20.4H_2_O) was obtained from Thermo Fisher Scientific. For the solution-exchange method, Na-MOR, the starting material, was mixed with fully saturated solutions of CsCl, SrCl_2_·6H_2_O, Cd(NO_3_)_2_·4H_2_O, and Pb(NO_3_)_2_ (from Daejung Chemicals and Metals) in a 1 g: 100 mL weight ratio. The mixture was stirred at 80 °C for 24 h in a closed system to minimize the loss of water. The solid was separated from the solution by vacuum filtration, and this cycle was repeated five times. The final products (Cs-, Sr-, Cd-, and Pb-MOR) were washed with distilled water and air-dried at room temperature for 24 h. Energy dispersive X-ray spectroscopy analysis (EDS, SUPRA25, Oxford Instruments) operating at 15 kV accelerating voltage at Pusan National University, Busan, Korea, was used to confirm that final products were almost fully exchanged, with the residual Na^+^ cation detected at less than 0.1%. To determine the amount of H_2_O molecules in the unit cell, Thermogravimetric analysis (TGA, Discovery SDT 650, TA Instruments) was performed at Kyungpook National University, Daegu, Korea. The samples were heated up to 1000 °C at a rate of 10 °C/min under a nitrogen atmosphere (Figure S1). The chemical analysis results are summarized in Table 1.

**Table 1:** EDS Chemical Composition of the Starting Material and Fully Exchanged Cs-, Sr-, Cd-, and Pb-Mordenite^a^

	Atomic percent (%)						
Sample	Element	1	2	3	Average	Weight percent of H_2_O (%)	Composition
Na-MOR	Na	4.22	4.17	4.27	4.22	10.8(1)%	Na_6.6_Al_6.6_Si_41.4_O_96_·20.4H_2_O
	Al	4.00	3.74	3.81	3.85		
	Si	25.42	23.10	23.71	24.08		
Cs-MOR	Cs	3.12	3.5	3.45	3.36	8.4(1)%	Cs_6.8_Al_6.8_Si_41.2_O_96_·19.4H_2_O
	Na	0.00	0.00	0.00	0.00		
	Al	3.20	3.47	3.50	3.39		
	Si	19.40	21.37	21.19	20.65		
Sr-MOR	Sr	1.41	1.41	1.36	1.39	11.6(1)%	Sr_3.3_Al_6.6_Si_41.4_O_96_·23H_2_O
	Na	0.00	0.00	0.00	0.00		
	Al	3.69	3.56	3.47	3.57		
	Si	23.03	22.08	21.52	22.21		
Cd-MOR	Cd	1.59	1.68	1.69	1.65	11.2(1)%	Cd_3.4_Al_6.8_Si_41.2_O_96_·22.8H_2_O
	Na	0.00	0.00	0.00	0.00		
	Al	3.60	3.67	3.72	3.66		
	Si	21.75	22.89	23.55	22.73		
Pb-MOR	Pb	1.50	2.00	1.62	1.71	9.6(1)%	Pb_3.2_Al_6.4_Si_41.6_O_96_·21H_2_O
	Na	0.00	0.00	0.00	0.00		
	Al	2.9	3.6	3.05	3.18		
	Si	18.81	24.71	19.99	21.17		

a Values are normalized based on 96 oxygen atoms per unit cell.

### Synchrotron X-ray Powder Diffraction

X-ray diffraction experiments were performed on a 3D (X-ray Scattering, XRS) beamline and 5A (Material Science XRS, MS-XRS) beamline in the Pohang Accelerator Laboratory (PAL). The X-ray from the bending magnet at the 3D beamline and the insertion device at the 5A beamline was monochromatized by cryogenically cooled silicon (111) double crystals, and sets of parallel slits were used to produce a wavelength of 0.6877(1) Å for 3D and 0.6926(1) Å for 5A. In both beamlines, a two-dimensional MAR345 imaging plate detector (100 μm pixel resolution, MarXperts) at 315 mm and 265 mm distance was used for XRD measurements, and the diffraction patterns were integrated with the DIOPTAS software.^21^ Detector geometries including sample to detector distance, and detector tilt based on incoming beam, were refined based on a CeO_2_ (NIST SRM 674b) powder diffraction pattern using DIOPTAS. The powder samples were loaded into a 0.5 mm Boron-Rich capillary (Charles Supper Company) and measured with an exposure time of 30 s, covering a 2θ range up to approximately 28°.

### CO_2_ Adsorption Isotherm

CO_2_ (purity 99.99%) adsorption isotherms were performed with a BELSORP-mini II (MicrotracBEL Corp.) at 298 K in the pressure range of 0–1 bar at Yonsei University, Seoul, Korea (Figure 6). Prior to the measurement, ∼ 300 mg of sample was loaded in a sample cell and dried by removing surface water under a dynamic vacuum at 353 K for 12 h.^22,23^ The amount of CO_2_ gas uptake was calculated from the Brunauer–Emmett–Teller method (BET).^24,25^
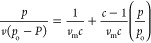
1

### Rietveld Structure Refinement

The changes in the unit cell constants were determined by whole pattern fitting using the GSAS suite of programs.^26^ The background curve was fitted with a Chebyshev polynomial with <30 coefficients, and the pseudo-Voigt profile function (Table S1) proposed by Thompson was used to model the observed Bragg reflection.^27^ The structural models of the Na-, Cs-, Sr-, Cd-, and Pb-MOR at ambient conditions were then established by Rietveld methods.^28,29^ As a starting model, we used the structure model of synthetic Na-mordenite. Given the strong pseudosymmetry reported by Simoncic and Armbruster, the measured diffraction peaks are indexed in the orthorhombic space group ***C****mcm*.^30^ The tetrahedral site was assumed to be statistically occupied by Si and Al atoms. The result of the chemical analysis was then used to set the Si/Al ratio to 0.86/0.14 at the disordered distribution of Si and Al atoms. Geometrical restraints were applied on the Si/Al disordered tetrahedra based on the set ratio of Si/Al; T–O (T = Si, Al) and O–O interatomic distances of the tetrahedra restrained to target values of 1.638 ± 0.001 and 2.676 ± 0.005 Å, respectively. All the isotropic displacement factors (*U*_iso_) were refined by grouping the framework tetrahedral atoms, the framework oxygen atoms, and the extra-framework cations, respectively, to minimize the number of parameters. The amounts of water molecules in the unit cell were calculated by using the result of Rietveld refinement with OW1, OW2, OW3, and OW4 multiplicities and occupancies. Using successive difference Fourier syntheses, the distributions of the extra-framework cations in the channel were found. The final convergence of the refinement was achieved by simultaneously varying all the background and profile parameters, scale factor, lattice constants, 2θ zero, and atomic positional and thermal displacement parameters. The parameters of the final refined models are summarized in Tables S1 and S2. Bond valence sum (BVS) was calculated based on the refined structural models.^31,32^ The average BVS values for Na^+^, Cs^+^, Sr^2+^, Cd^2+^, and Pb^2+^ are 0.68, 0.83, 2.13, 1.14, and 1.17, respectively (Table S2). Both Cd^2+^ and Pb^2+^ cations indicated a significant deviation from the formal valence. Although Cd^2+^ ions have the same distribution as Sr^2+^ ions, they are under-bonded with the structure due to a smaller ionic radius than Sr^2+^ ions. Pb^2+^ ions have a similar ionic radius to Sr^2+^ ions but are under-bonded, likely due to a lower number of water molecules (Pb-MOR: Pb_3.2_Al_6.4_Si_41.6_O_96_·21H_2_O; Sr-MOR: Sr_3.3_Al_6.6_Si_41.4_O_96_·23H_2_O) in the unit cell. Thus, the CO_2_ uptake is explained by a complex correlation with the size and distribution of cations within the 8MR and electronegativity.

## Results and Discussion

### Changes of X-ray Diffraction Patterns for Cation Exchanged MORs

**Figure 2:**
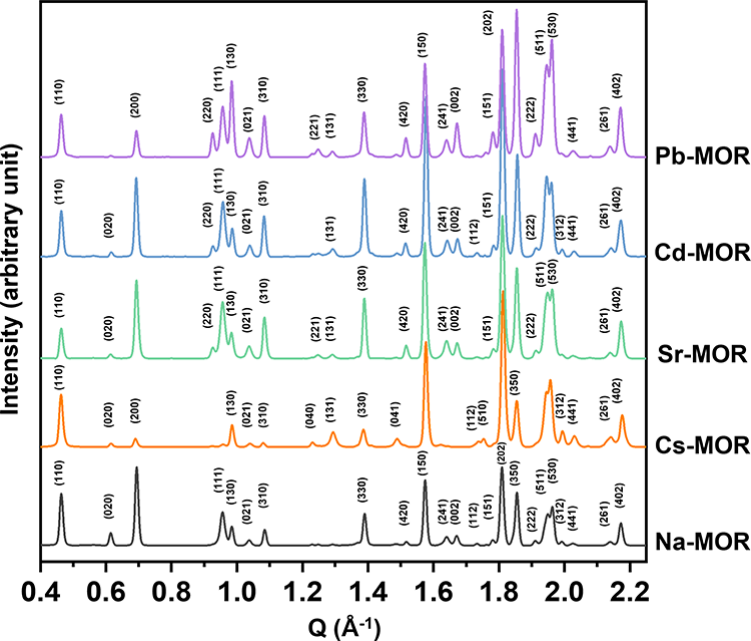
Synchrotron X-ray powder diffraction patterns measured for Na-MOR, Cs-MOR, Sr-MOR, Cd-MOR, and Pb-MOR under ambient conditions. Several Bragg reflections are marked to compare the changes in their relative intensities.

The synchrotron powder X-ray diffraction (XRD) patterns of all of the samples were indexed in the orthorhombic ***C****mcm* space group under ambient conditions, as shown in Figure 2. The changes in relative intensity are dependent on the exchangeable cations.^33^ For Cs-MOR compared to Na-MOR, the intensities of the (131), (150), and (202) reflections significantly increased, while those of the (200), (111), and (310) reflections decreased. These intensity changes are associated with the specific structural arrangement of the heavier Cs^+^ ions in the channels and match closely with results reported by Dimowa et al.^34^ In the divalent-cation forms, both Sr- and Cd-MOR exhibited similar XRD patterns with specific differences compared to Na-MOR. In Cd-MOR, there was an increase in the (330) reflection and an inversion in the intensity of the (511) and (530) reflections. In Pb-MOR, an inversion of the (111) and (130) reflections was observed, unlike other divalent-cation forms. Note that a (220) reflection, absent in the monovalent-cation forms (Na- and Cs-MOR), was represented in the divalent-cation forms (Sr-, Cd-, and Pb-MOR). These alterations in atomic distribution mostly occur on the *ab*-plane, with slight changes in the unit cell constants.

### Effects of Electronegativity of Cation on the Mordenite Structure

**Figure 3:**
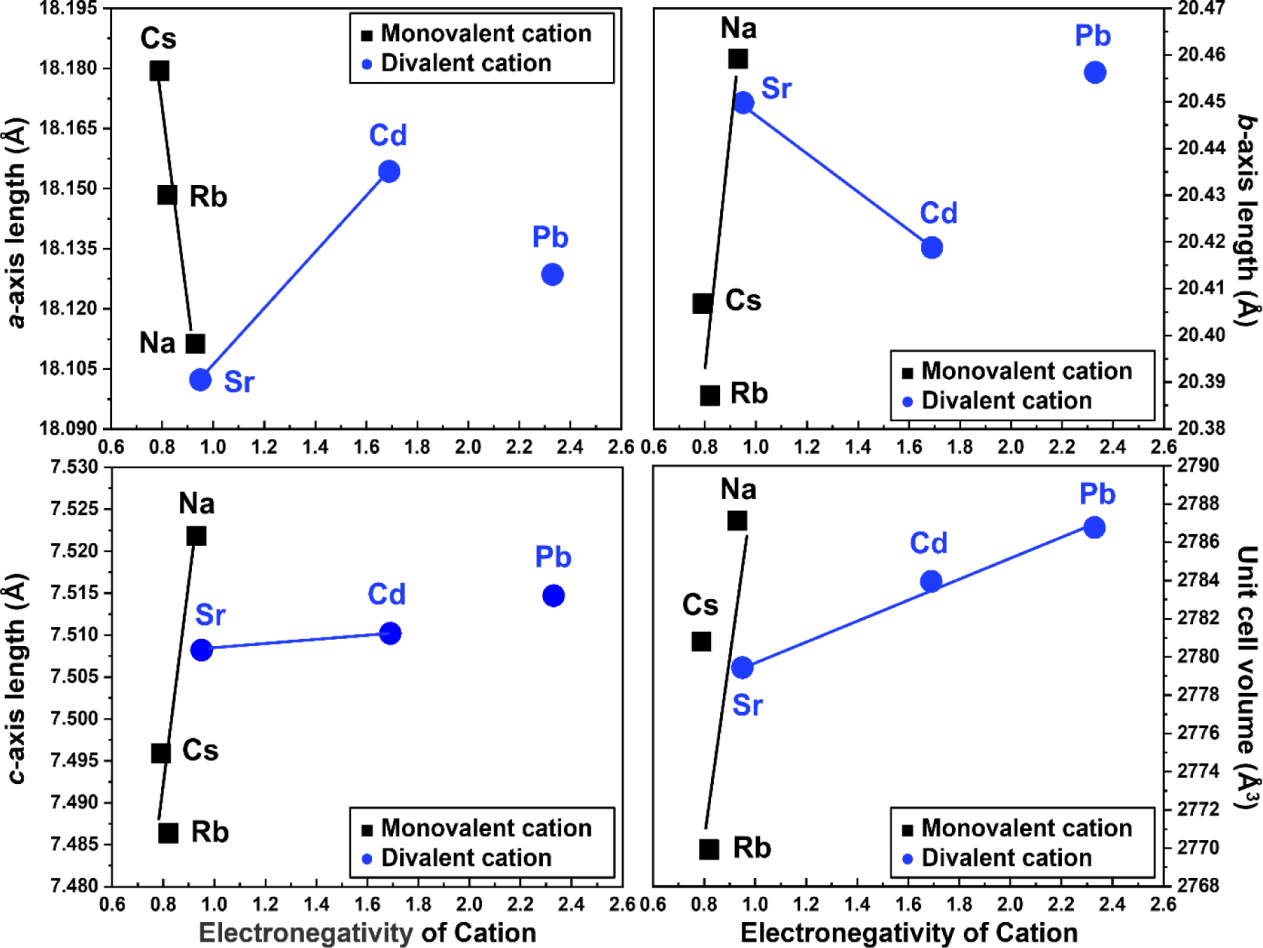
Correlation between the refined *a*-axis, *b*-axis, *c*-axis, and unit cell volumes of the cation-exchanged mordenites and the electronegativity of the extra-framework cation is explored. Data for Rb-MOR are from the work of Itabashi et al. (2007).^35^ Pb-MOR deviates from the trends observed in other divalent-MORs groups.

Detailed changes in the unit cell parameters for the various MORs were derived by using the whole pattern fitting method within the GSAS suite of programs, as shown in Figure 3 and Figure S2. For comparison, the Rb-MOR model, a monovalent-cation form, was cited from Itabashi et al.^35^ The *a*-axis lengths of Cs-, Rb-, and Na-MOR exhibited a sharp decrease in the range of 18.11 (5)–18.18(5) Å as the electronegativity of the cation increased slightly (Figure 3). Conversely, the *b*-axis length increased significantly within the range of 20.38(5)–20.46(5) Å and the *c*-axis length remained nearly constant within the range of 7.485(5)–7.525(5) Å.

In the divalent-cation forms such as Sr- and Cd-MOR, the *a*-axis length increased, while the *b*-axis length decreased with increasing cation electronegativity. The *c*-axis length of the divalent-cation forms did not show significant changes, exhibiting trends opposite those of the monovalent-cation forms. This behavior can be explained by the deformation of the lattice parameters primarily on the *ab*-plane, consistent with powder X-ray diffraction patterns. Pb-MOR showed distinct changes in lattice parameters, unlike other divalent-cation-exchanged MORs. Consequently, the increase in the unit cell volume for Cs-, Rb-, and Na-MOR was more than 16 times that of Sr-, Cd-, and Pb-MOR with increasing cation electronegativity.

**Figure 4:**
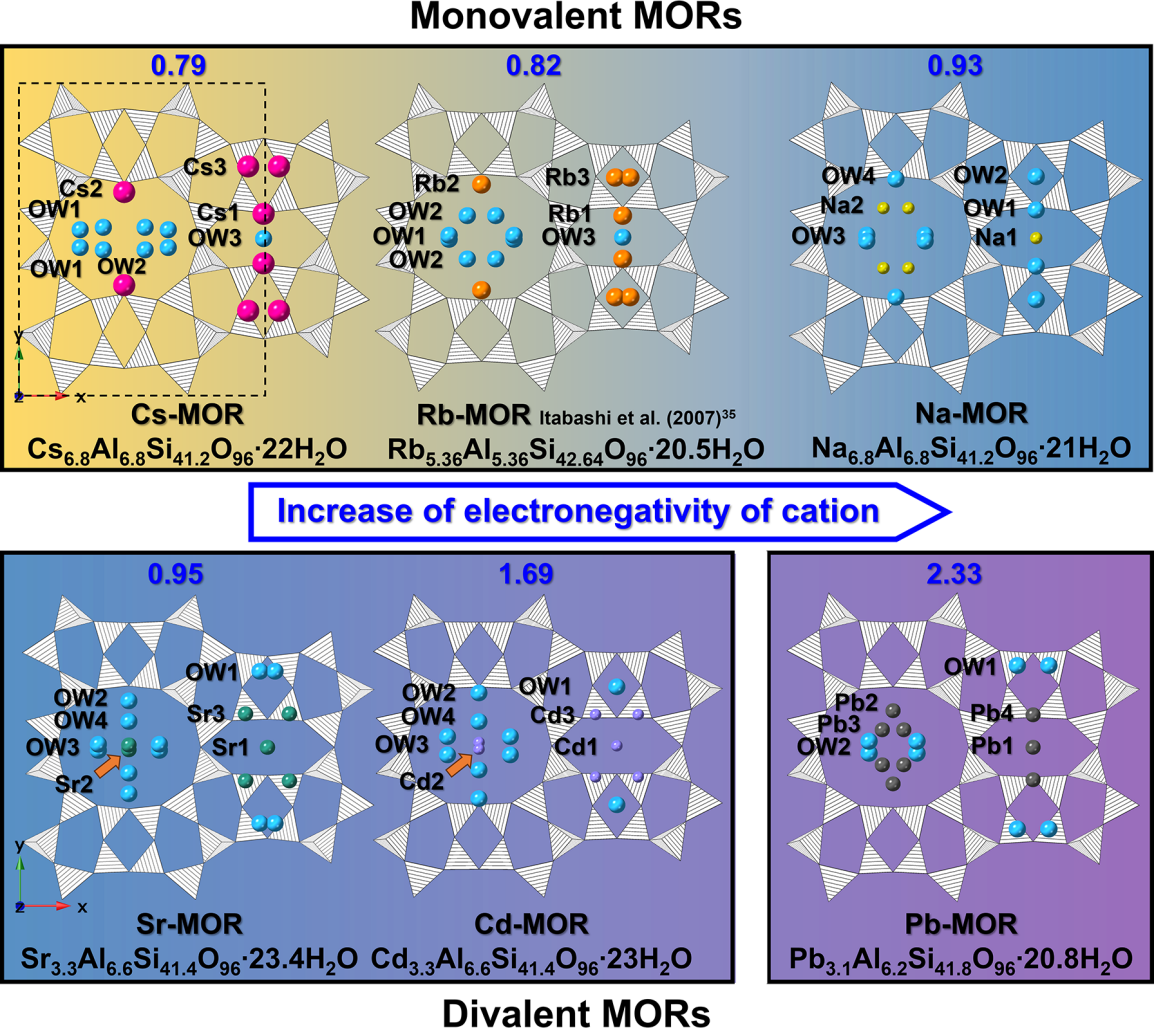
Polyhedral representations of monovalent-exchanged mordenites (Cs-, Rb-, and Na-MOR) and divalent-exchanged mordenites (Sr-, Cd-, and Pb-MOR) showing the respective structural and chemical changes with increasing electronegativity at ambient viewed along the (001) direction. Striped black tetrahedra illustrate a disordered distribution of Si (Al) atoms in the framework. Blue, magenta, orange, yellow, green, violet, and black circles represent H_2_O molecules, Cs^+^, Rb^+^, Na^+^, Sr^2+^, Cd^2+^, and Pb^2+^ extra-framework cations, respectively.

To gain detailed insights into structural changes with exchangeable cations, structural models for the MORs were derived using Rietveld refinement (Figure S3). All models were oriented along the *ab*-plane to visualize changes in extra-framework species (EFS) within the channels (Figure 4). The chemical compositions of all samples used for structural analysis are summarized in Table 1. Refined fractional coordinates derived from Rietveld refinement are provided in Table S1.

In Na-MOR, two cation sites and four water molecule sites were identified in the channel. Na^+^ ions are located at the center of the 8MRc (Na1 site) and in the 12MRc (Na2 site), while water molecules are found near the 8MRb (OW1, OW2, and OW4 sites) and in the 12MRc (OW3 site). The Na1 site, exhibiting eight-coordinate bonding with six framework oxygens and two water molecules, had an occupancy of about 35%. The Na2 site, with four-coordinate bonding involving one framework oxygen and three water molecules, had an occupancy of about 65%. The chemical formula of Na-MOR, calculated using the Rietveld refinement, was Na_6.8_Al_6.8_Si_41.2_O_96_·21.8H_2_O, indicating the presence of 21.8 water molecules per unit cell, consistent with chemical analysis results.

After Cs^+^ exchange (lower electronegativity than Cs^+^ = 0.79; Na^+^ = 0.93 by Pauling’s scale), differences in EFS sites were observed.^36^ Cs^+^ ions were mainly located at the 8MRb (Cs1, Cs2, and Cs3 sites), while water molecule sites were in the 12MRc (OW1 and OW2 sites) and at the center of the 8MRc (OW3 site). This observation aligns with previous reports showing Cs^+^ has a strong affinity for 8MR building units regardless of the zeolite structure.^37^ The Cs1 site (50% occupancy) exhibited nine-coordinate bonding with eight framework oxygens and one water molecule, while the Cs2 site (38% occupancy) had 12-coordinate bonding with eight framework oxygens and four water molecules. The Cs3 site (12% occupancy) exhibited eight-coordinate bonding with the framework oxygens. The simultaneous occupation of Cs1 and Cs3, or Cs2 and Cs3 sites, was excluded due to strong electrostatic repulsion between these Cs^+^ cations.^35^ Rb-MOR, like Cs-MOR, showed Rb^+^ ions exclusively at the 8MRb (Rb1, Rb2, and Rb3 sites), with water molecules located in the 12MRc (OW1 and OW2 sites) and 8MRc (OW3 site). Compared to Na^+^ sites, Cs^+^ and Rb^+^ sites exhibited higher dependence on zeolite structure, attributed to differences in electronegativity and hydration energy.^38^ Cations like Cs^+^ and Rb^+^, with lower hydration energies (−245(5) and −285(10) kJ/mol respectively), compared to Na^+^ (−385(20) kJ/mol), can be readily dehydrated before coordination in the 8MR units.^37,39,40^

For Sr-MOR, the Sr1 site (28% occupancy) had six-coordinated and the Sr3 site (36% occupancy) had seven-coordinated bonding with framework oxygens. The Sr2 site (36% occupancy) exhibited 8-fold coordination, surrounded by water molecules at the center of the 12MRc. Cd^2+^ ions and water molecule positions in Cd-MOR were similar to those in the Sr-MOR model. The Cd1 site (23% occupancy) had six-coordinated and the Cd3 site (41% occupancy) had seven-coordinated bonding with framework oxygens, while the Cd2 site (36% occupancy) was 8-fold coordinated and surrounded by water molecules in the 12MRc.

**Figure 5:**
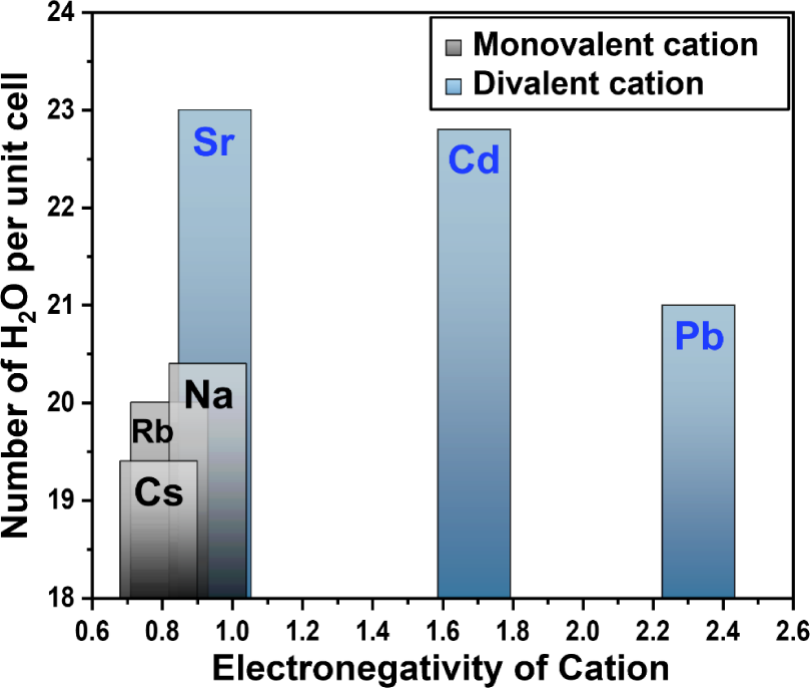
Number of water molecules per unit cell in Cs-, Rb-, Na-, Sr-, Cd-, and Pb-MOR as the electronegativity of the extra-framework cations.

Unlike the monovalent-cation forms, divalent-cation forms showed significantly different cation positions due to differences in hydration energies and electronegativity.^41^ For Pb-MOR, which had four cation sites and two water molecule sites in the channel, the Pb1 (38% occupancy) and Pb4 (3% occupancy) sites had six-coordinated and eight-coordinated bonding with framework oxygens, respectively. The Pb2 site (33% occupancy) exhibited five-coordinate bonding with three framework oxygens and two water molecules, while the Pb3 site (26% occupancy) had five-coordinate bonding with five water molecules. Despite the relatively high hydration energy of Pb^2+^ ions (−1,345(80) kJ/mol), partially hydrated Pb^2+^ ions were observed near the 12MRc wall. Pb^2+^ ions showed a strong affinity for the electron cloud of the framework oxygen atoms in the 12MRc due to their higher electronegativity (2.33) compared to Si (1.90) and Al (1.61) atoms,^42^ reducing water molecule content compared to other divalent-cations (Figure 5).

### Trend on CO_2_ Gas Adsorption for Cation Exchanged MORs

**Figure 6:**
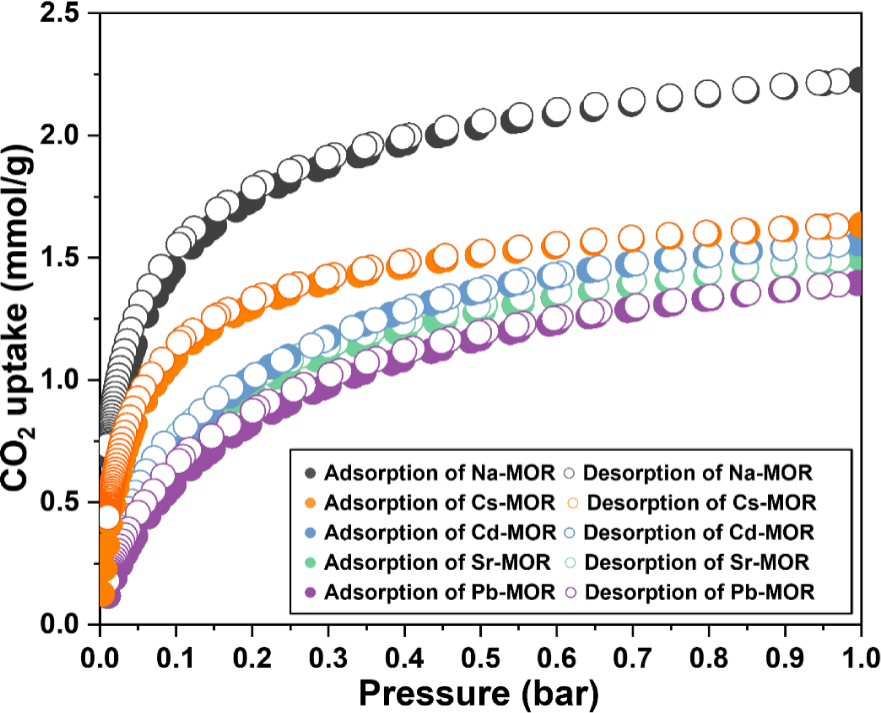
CO_2_ adsorption and desorption isotherms at 298 K up to 1 bar on Na-, Cs-, Cd-, Sr-, and Pb-MOR. Filled symbols represent adsorption; open symbols represent desorption.

**Figure 7:**
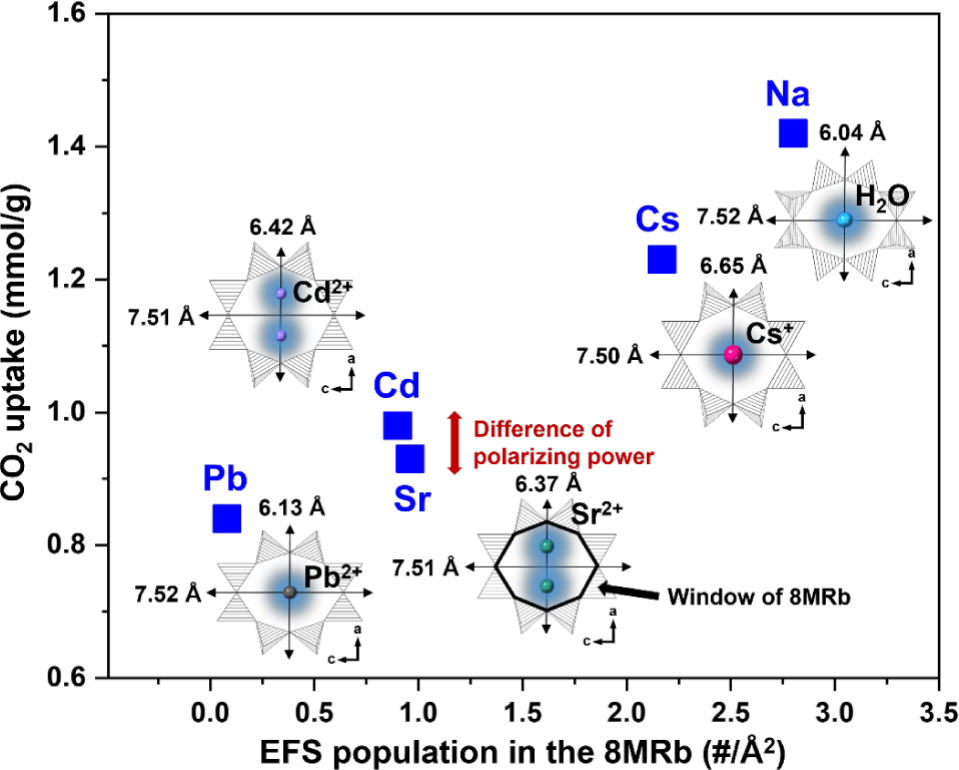
Changes in CO_2_ uptake (mmol/g) of the cation-exchanged mordenites as a function of the number of extra-framework species per window area (#/Å^2^) of the 8-membered ring along the *b*-axis (8MRb). Details of the respective chemical changes observed in the window of the 8MRb were associated with amounts of CO_2_ uptake. The EFS in the 8MRb is highlighted in blue.

The CO_2_ gas adsorption for MORs was obtained from CO_2_ isotherms measured at 25 °C (Figure 6). According to Fu et al., the primary site for CO_2_ adsorption of Na-mordenite is located at the 8MRb, and its size is crucial for performance.^43^ To explore the relation between the 8MRb and CO_2_ adsorption, the distribution of EFS in the channels and channel size was determined using the Rietveld method. The degree of EFS population was calculated as the number of EFS per unit cell divided by the window area in the 8MRb (#/Å^2^). It was found that CO_2_ uptake tends to increase with the degree of EFS population in the 8MRb because of the effective interaction with CO_2_ (Figure 7). The highest CO_2_ uptake was 1.42 mmol/g for Na-MOR, with water molecules in the center of the 8MRb. The presence of the water molecules induces the electrostatic interactions with CO_2_ in the 8MRb due to the highly polar nature of the water molecules and influences the CO_2_ adsorption performance.^44^ Cation-exchanged MORs showed decreasing CO_2_ uptake in the following order: Cs-MOR (1.23 mmol/g), Cd-MOR (0.98 mmol/g), Sr-MOR (0.93 mmol/g), and Pb-MOR (0.84 mmol/g). The results indicate that the presence of a cation in the 8MRb decreases CO_2_ adsorption compared to water molecules, and the nature of the cation acts as a dominant factor in adsorption. The strength of the basic sites of a framework increases with lower electronegativity of the cation because the negative charge on the framework oxygen atoms increases with the decrease in electronegativity of the neighboring cation.^7^ Therefore, stronger basic sites can interact more strongly with the acid CO_2_, increasing its adsorption performance. However, as the radius of the cation increases (Sr^2+^ > Cd^2+^), the polarizing power of the cation decreases, resulting in a weaker interaction with CO_2_.^45^ In summary, the CO_2_ adsorption capacity for the mordenite framework can be attributed to a complex balance between the population and polarizing powers of the cation in 8MRb as the basic site.

## Conclusions

In this study, we have demonstrated the structural changes in Na-MOR after ion exchange with both mono- and divalent-cations. The structures of Cs- and Rb-MOR revealed that these cations are located exclusively at the 8MRb sites, while water molecules are primarily situated in the 12MRc channels. This is because Cs^+^ and Rb^+^ ions have lower hydration energies (−245(5) and −285(10) kJ/mol, respectively) compared to other alkali cations (Na^+^ has a hydration energy of −385(20) kJ/mol), allowing them to shed the surrounding H_2_O molecules more readily before being coordinated in the 8MRb. In contrast, Na^+^ ions are positioned at the center of the 8MRc and in the 12MRc, with water molecules located near the 8MRb and in the 12MRc. For Sr- and Cd-MOR, the high hydration energies of Sr^2+^ and Cd^2+^ (−1,385(5) and −1,575(180) kJ/mol, respectively) relative to Na^+^ ions result in these hydrated divalent ions being situated at the center of the 12MRc. For Pb-MOR, Pb^2+^ ions show a strong affinity for the electron cloud of the framework oxygen atoms in the 12MRc due to their higher electronegativity (2.33) compared to Si (1.90) and Al (1.61) atoms, leading to Pb^2+^ ions being primarily located near the walls of the 12MRc. We observed that the changes in unit cell parameters and the number of water molecules per unit cell are influenced by the electronegativities of the cation.

Additionally, the CO_2_ uptake for the MOR is influenced by a complex balance between the population and polarizing powers of the cation in 8MRb as the basic site. Understanding how different cations impact the structure and adsorption capacities is crucial for developing advanced ion-exchanged zeolites tailored for specific pollutant capture needs. The detailed structural analysis of fully exchanged MORs can provide potential applications in large scale systems for future studies. Also, it may be fundamental data for applications involving temperature- and pressure-induced capture and the comparative stability of cation-exchanged mordenite.

## Supporting Information Available

Thermogravimetric analysis results; LeBail refinement and Rietveld refinement fit of the structural models; detailed parameters of the final refined models (PDF)
