# Supplementary material for: Relationship Between Electronegativity of the Extra-Framework Cations and Adsorption Capacity for CO2 Gas on Mordenite Framework
Source: Inorg Chem. 2024 Dec 26;64(2):903–10. doi: 10.1021/acs.inorgchem.4c04062 (PMC11752500; doi:10.1021/acs.inorgchem.4c04062)
Supplement: Supplementary file 1 — ic4c04062_si_001.pdf [file ic4c04062_si_001.pdf]

Supporting information

Relationship between Electronegativity of the Extra-Framework Cations and Adsorption Capacity for CO_2 Gas on Mordenite framework

Soojin Lee,[1] Hyunseung Lee,[1] Huijeong Hwang,[2] Donghoon Seoung,[3] Hyeonsu Kim,[3] Pyosang Kim,[3] and Yongmoon Lee[1,4,]*

[1]Department of Geological Sciences, Pusan National University, Busan 46241, Korea; [2]School of Environment and Energy Engineering, Gwangju Institute of Science and Technology, Gwangju 61005, Korea; [3]Department of Earth and Environmental Sciences, Chonnam National University, Gwangju 61186, Korea; [4]Institute for Future Earth Environment, Pusan National University, Busan 46241, Korea

*Corresponding author. E-mail: lym1229@pusan.ac.kr

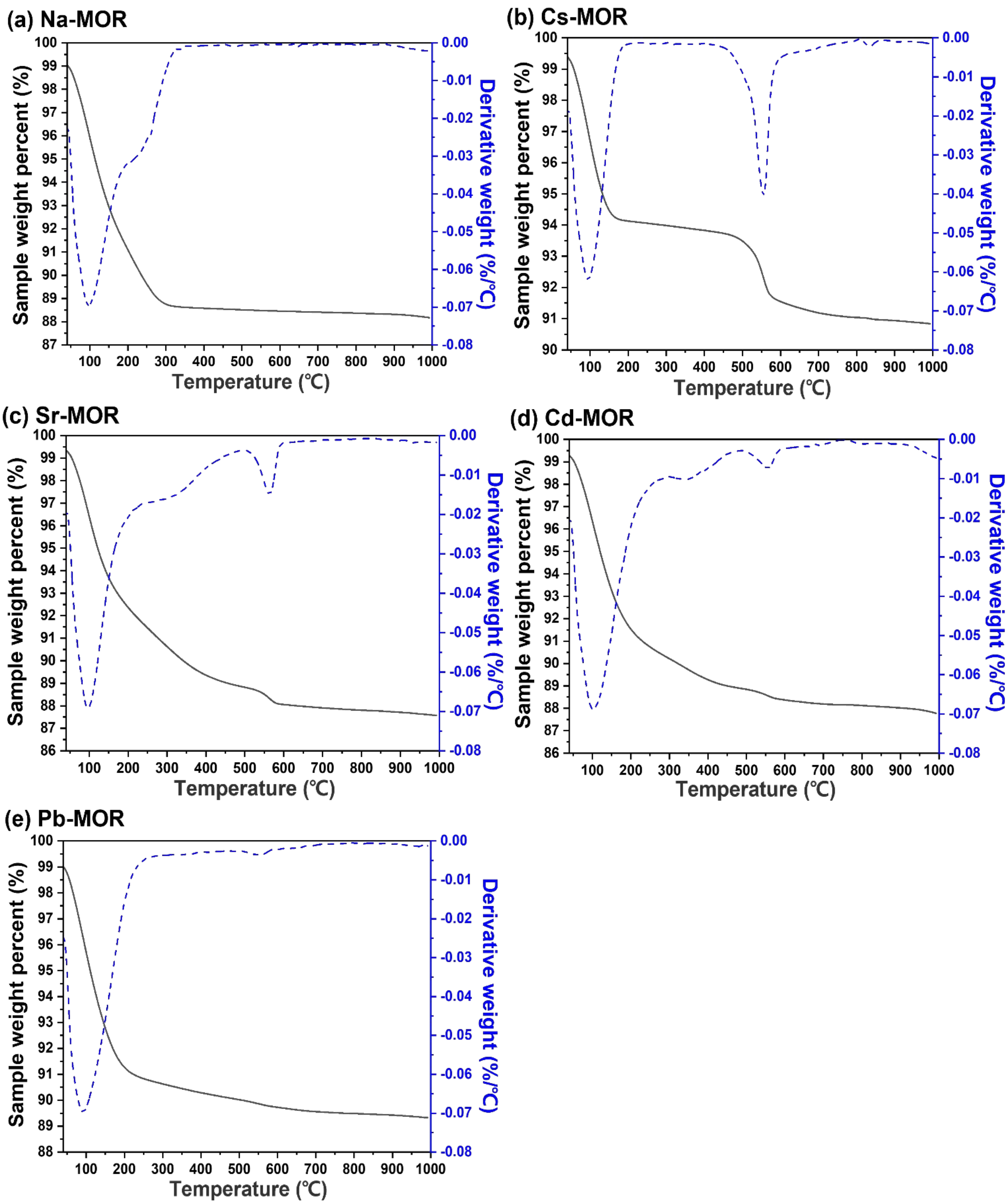

Fig. S1. Thermogravimetric analysis results of (a) Na-MOR, (b) Cs-MOR, (c) Sr-MOR, (d) Cd-MOR, and (e) Pb-MOR.

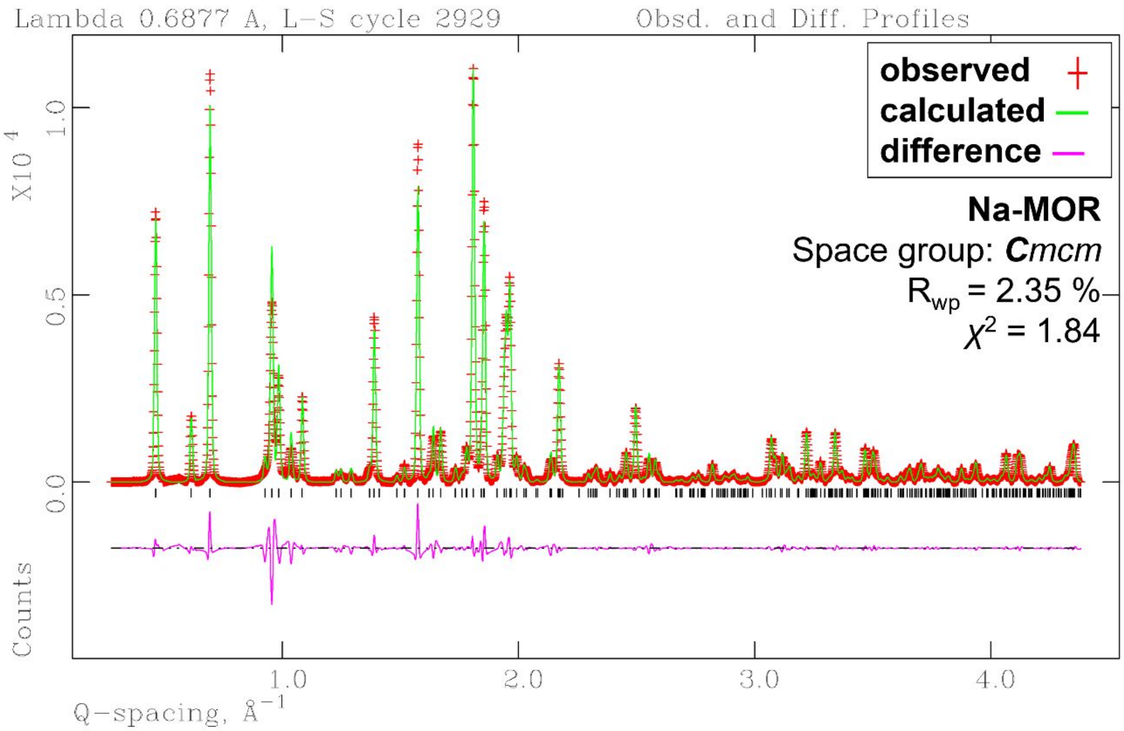
Lambda 0.6877 A, L-S cycle 2929
Obsd. and Diff. Profiles
observed
calculated
difference
Na-MOR
Space group: Cmcm
R_wp = 2.35 %
χ^2 = 1.84
X10 4
Counts
1.0
0.5
0.0
1.0
2.0
3.0
4.0
Q-spacing, Å^-1

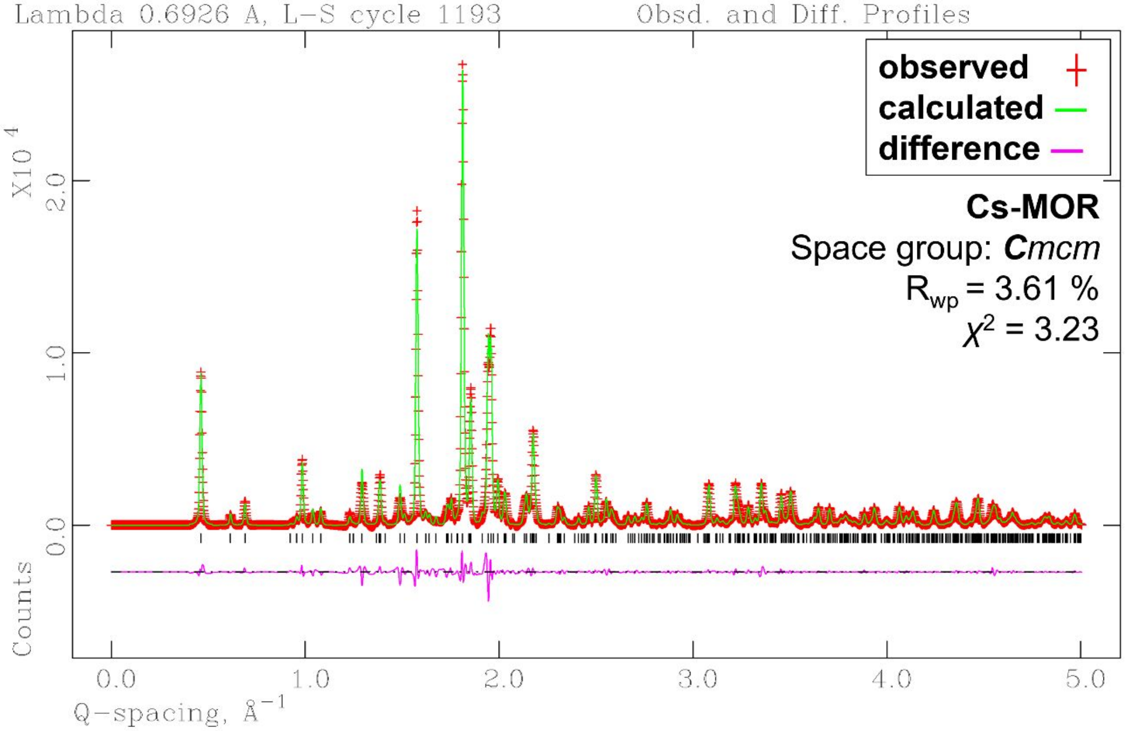
Lambda 0.6926 A, L-S cycle 1193
Obsd. and Diff. Profiles
observed
calculated
difference
Cs-MOR
Space group: Cmcm
R_wp = 3.61 %
χ^2 = 3.23
X10 4
Counts
2.0
1.0
0.0
0.0
1.0
2.0
3.0
4.0
5.0
Q-spacing, Å^-1

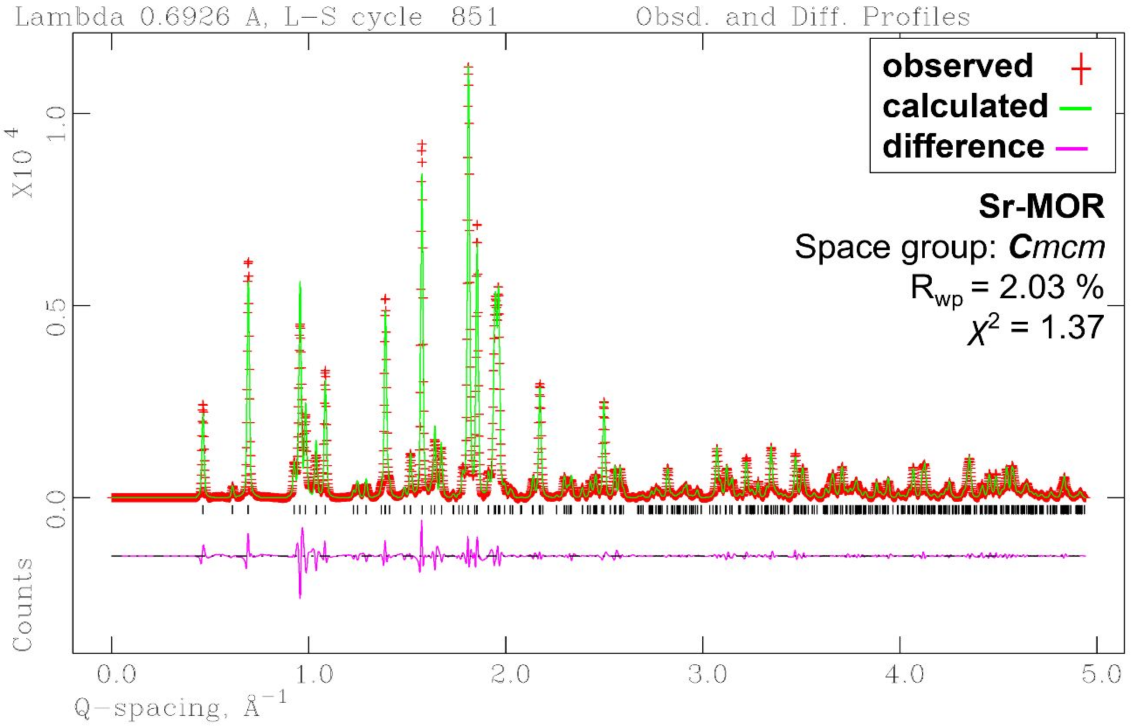
Lambda 0.6926 A, L-S cycle 851
Obsd. and Diff. Profiles
observed
calculated
difference
Sr-MOR
Space group: Cmcm
R_wp = 2.03 %
χ^2 = 1.37
X10 4
Counts
Q-spacing, Å^-1

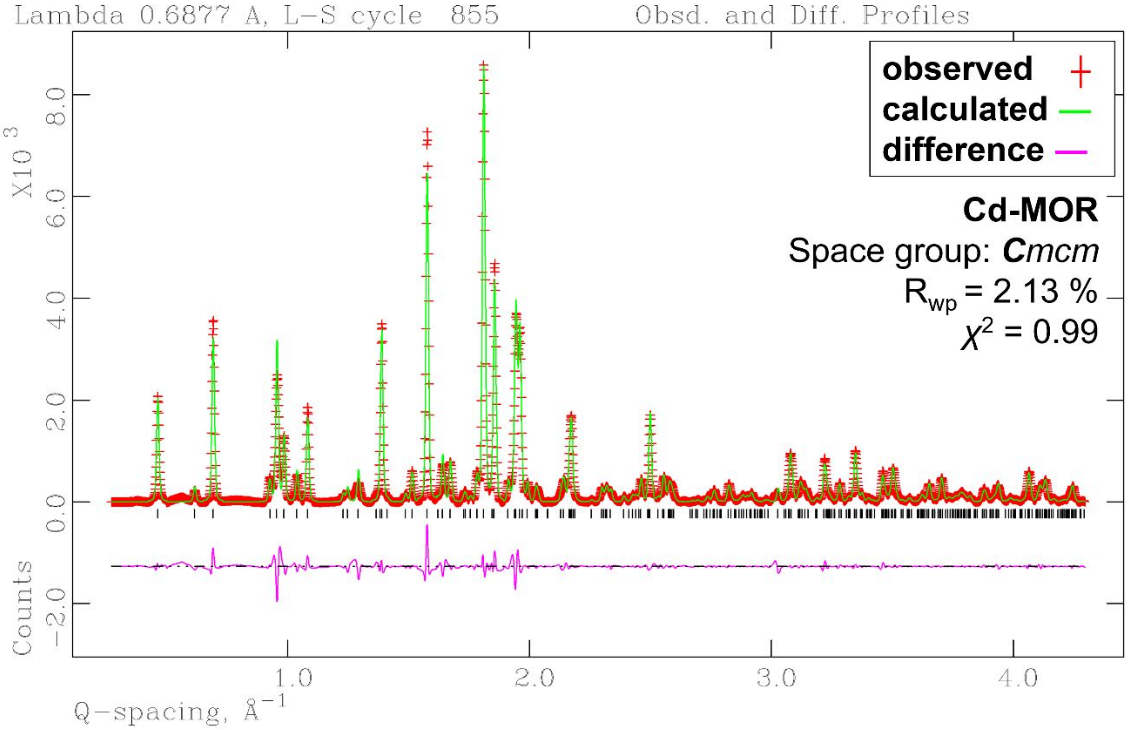
Lambda 0.6877 A, L-S cycle 855
Obsd. and Diff. Profiles
observed
calculated
difference
Cd-MOR
Space group: Cmcm
R_wp = 2.13 %
χ^2 = 0.99
X10 3
Counts
Q-spacing, Å^-1

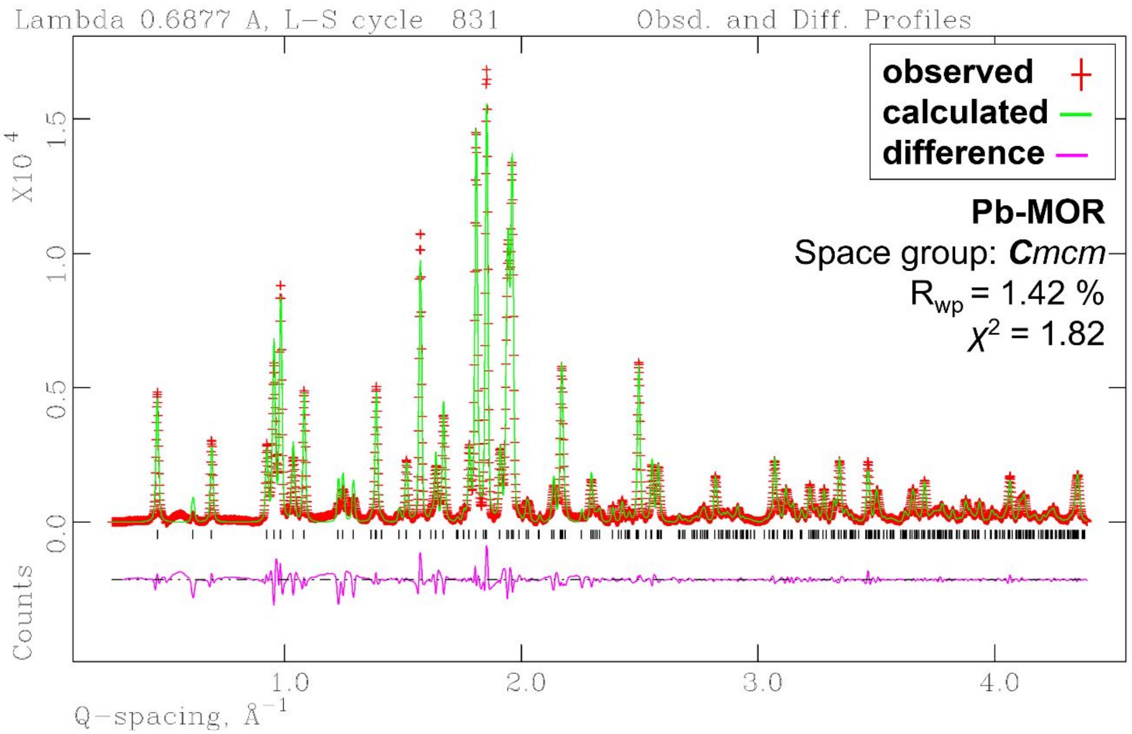

Fig. S2. LeBail refinement fits of the structural models of the Na-, Cs-, Sr-, Cd-, and Pb-mordenite at ambient using synchrotron X-ray powder diffraction data. Red point represents the observed data and green line represents the calculated data. The sets of tic marks below the data indicate the positions of the allowed reflections. The lower magenta curves represent the difference between observed and calculated profiles (Iobs - Icalc) plotted on the same scale as the observed data.

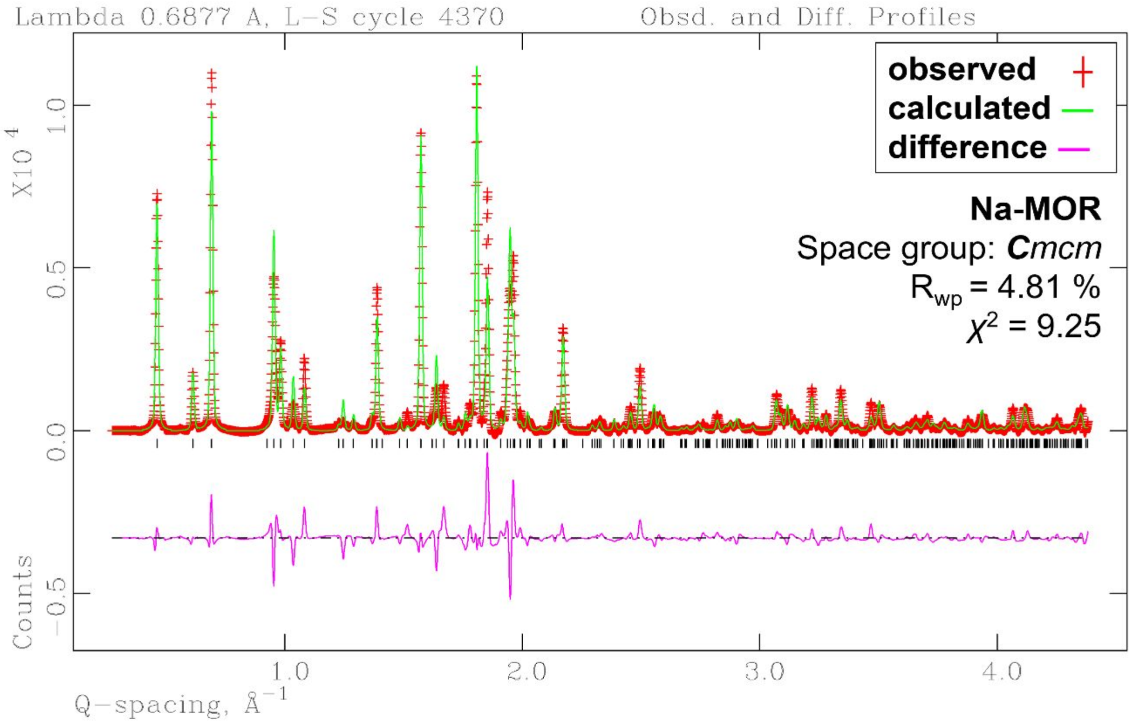
Lambda 0.6877 A, L-S cycle 4370
Obsd. and Diff. Profiles
observed
calculated
difference
Na-MOR
Space group: Cmcm
R_wp = 4.81 %
χ^2 = 9.25
X10 4
Counts
Q-spacing, Å^-1

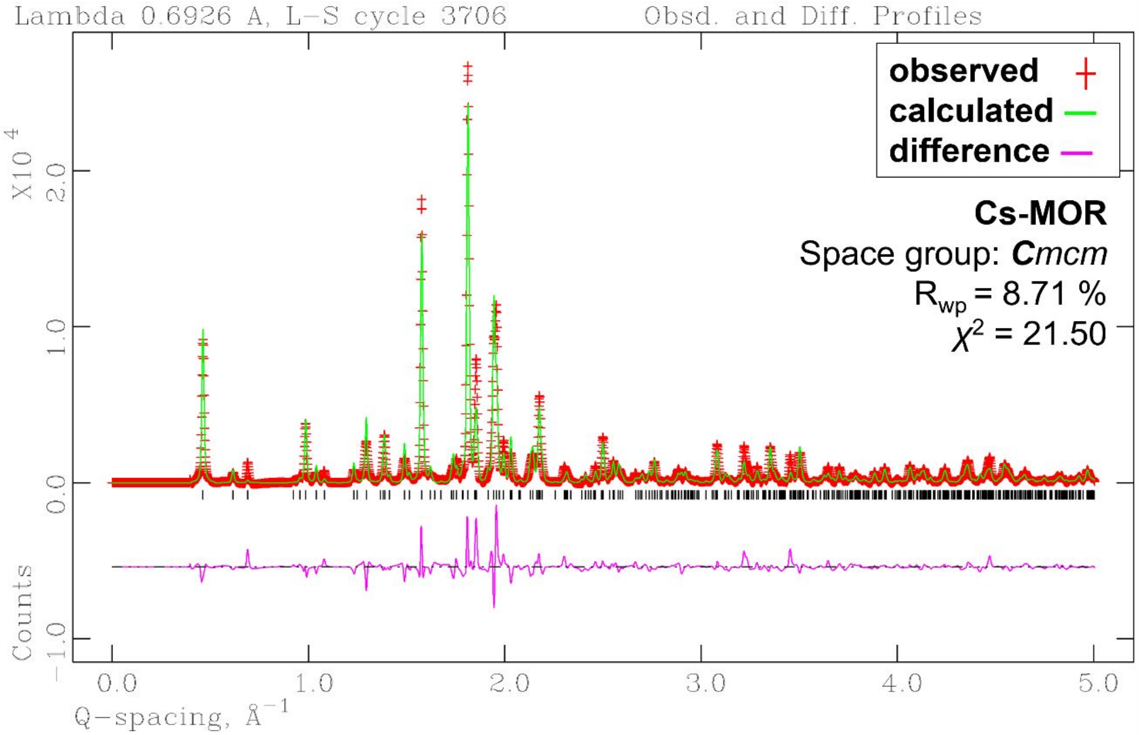
Lambda 0.6926 A, L-S cycle 3706
Obsd. and Diff. Profiles
observed
calculated
difference
Cs-MOR
Space group: Cmcm
R_wp = 8.71 %
χ^2 = 21.50
X10 4
Counts
Q-spacing, Å^-1

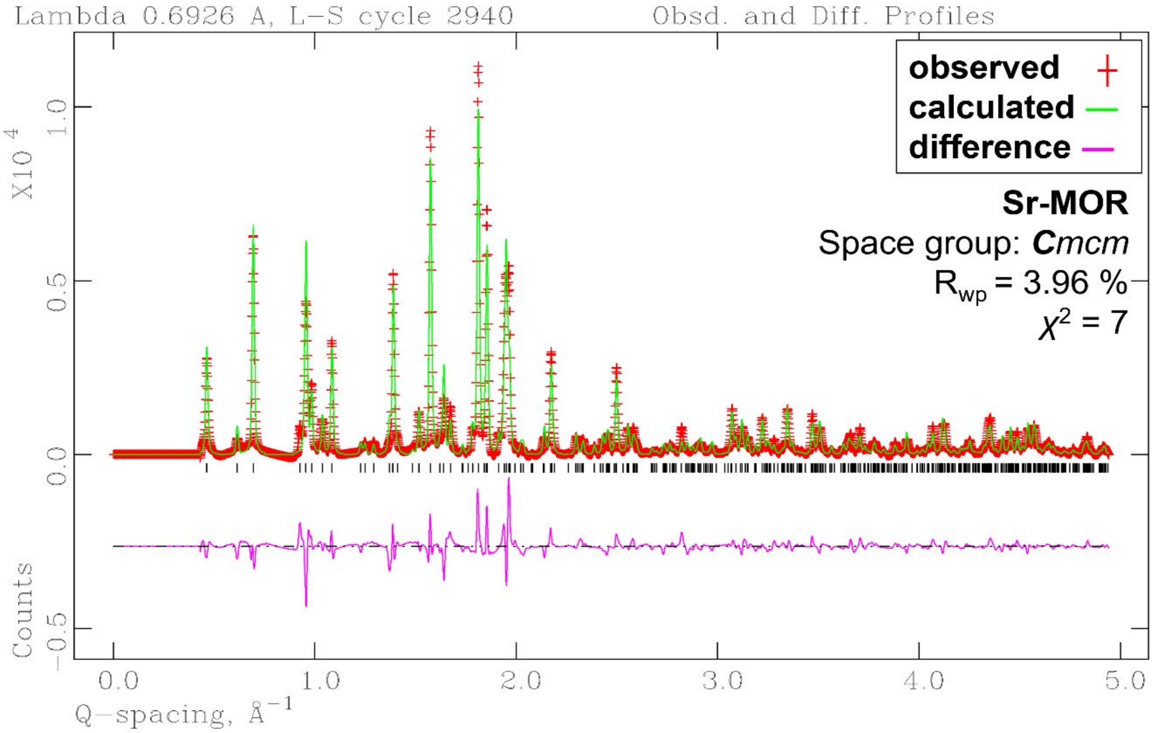
Lambda 0.6926 A, L-S cycle 2940
Obsd. and Diff. Profiles
observed
calculated
difference
Sr-MOR
Space group: Cmcm
R$_{wp}$ = 3.96 %
χ^2 = 7
X10 4
Counts
Q-spacing, Å^{-1}

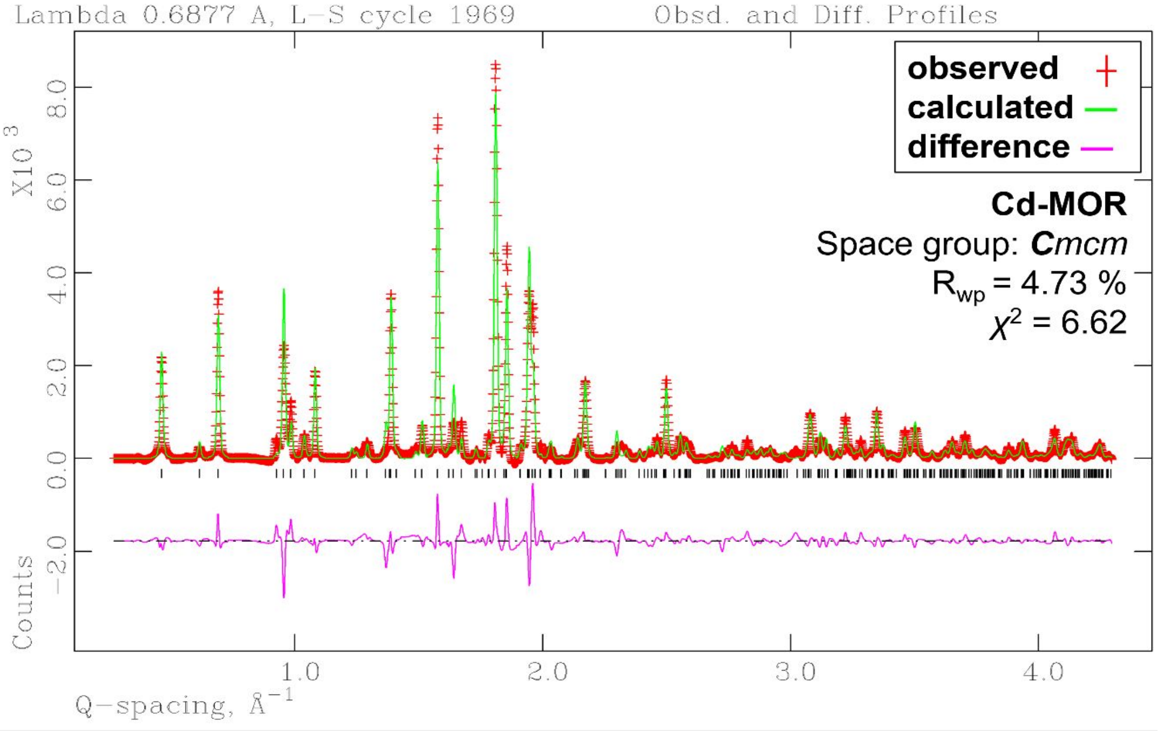
Lambda 0.6877 A, L-S cycle 1969
Obsd. and Diff. Profiles
observed
calculated
difference
Cd-MOR
Space group: Cmcm
R$_{wp}$ = 4.73 %
χ^2 = 6.62
X10 3
Counts
Q-spacing, Å^{-1}

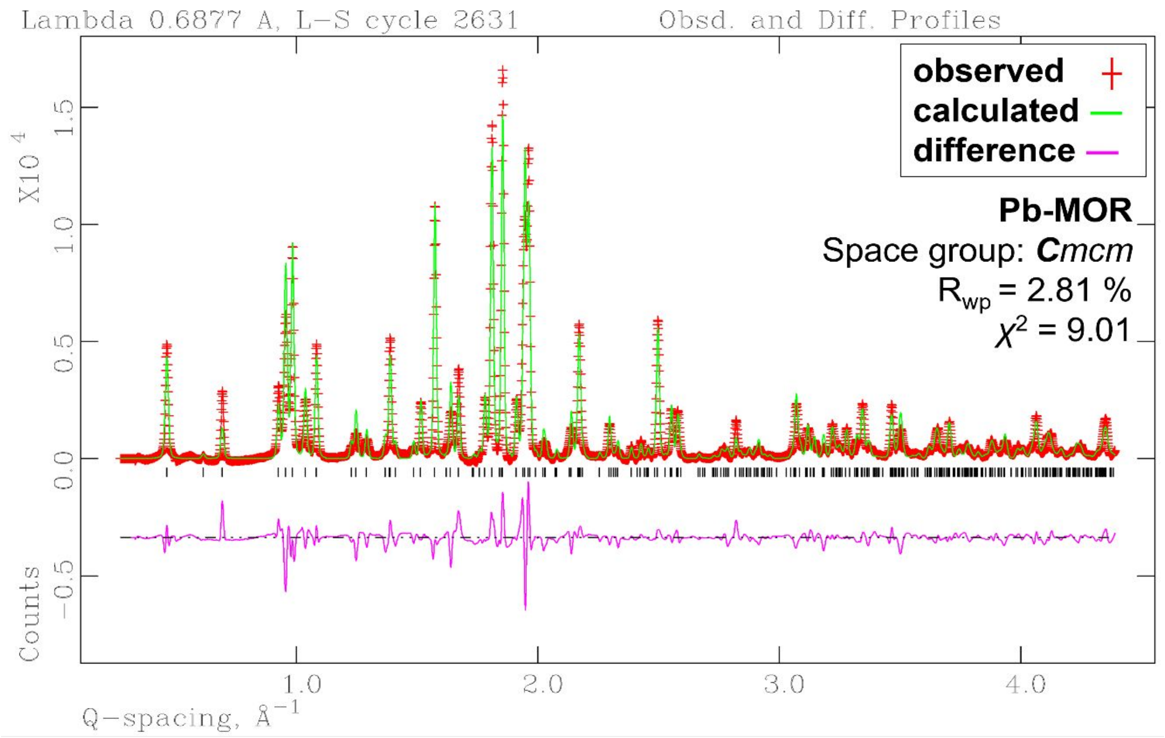

Fig. S3. Rietveld refinement fits of the structural models of the Na-, Cs-, Sr-, Cd-, and Pb-mordenite at ambient using synchrotron X-ray powder diffraction data. Red point represents the observed data. The green continuous lines through the sets of points are the calculated profiles from the structure refinements summarized in Table S1-2. The sets of tic marks below the data indicate the positions of the allowed reflections. The lower magenta curves represent the difference between observed and calculated profiles (Iobs - Icalc) plotted on the same scale as the observed data. According to Simoncic and Armbruster, domains of the entire ***Cmcm*** framework structure are reproduced by a non-crystallographic (001) mirror plane at z=0 and z=1/2. Diffuse scattering due to these structure defects is restricted to planes with Bragg indices *l*=2n+1 (e.g., 111, 311, 511). Thus, the intensity of a (511) reflection of all samples significantly decreased.

Table S1. Refined fractional coordinates for Na-, Cs-, Sr-, Cd-, and Pb-MOR under ambient condition.[a]

		Na-MOR	Cs-MOR	Sr-MOR	Cd-MOR	Pb-MOR
Space Group		***Cmcm***	***Cmcm***	***Cmcm***	***Cmcm***	***Cmcm***
$_{w}R_p$ (%), χ^2		4.81, 9.25	8.71, 21.5	3.96, 7	4.73, 6.62	2.81, 9.01
Chemical composition		$Na_{6.6}Al_{6.6}Si_{41.4}O_{96}$ $\cdot 20.4H_2O$	$Cs_{6.8}Al_{6.8}Si_{41.2}O_{96}$ $\cdot 19.4H_2O$	$Sr_{3.3}Al_{6.6}Si_{41.4}O_{96}\cdot$ $23H_2O$	$Cd_{3.4}Al_{6.8}Si_{41.2}O_{96}\cdot$ $22.8H_2O$	$Pb_{3.2}Al_{6.4}Si_{41.6}O_{96}\cdot$ $21H_2O$
Refined unit cell composition		$Na_{6.8}Al_{6.8}Si_{41.2}O_{96}$ $\cdot 21H_2O$	$Cs_{6.8}Al_{6.8}Si_{41.2}O_{96}$ $\cdot 22H_2O$	$Sr_{3.3}Al_{6.6}Si_{41.4}O_{96}$ $\cdot 23.4H_2O$	$Cd_{3.3}Al_{6.6}Si_{41.4}O_{96}$ $\cdot 22.9H_2O$	$Pb_{3.1}Al_{6.2}Si_{41.8}O_{96}$ $\cdot 20.8(H_2O)$
Pseudo-Voigt profile function parameters		GU: 149(33) GV: -23(4) GW: 8(1)	GU: 208(56) GV: -23(12) GW: 8(1)	GU: 362(50) GV: -83(12) GW: 11(1)	GU: 660(84) GV: -111(16) GW: 12(1)	GU: 928(75) GV: -171(16) GW: 15(1)
cell parameters	*a*	18.1113(16)	18.1794(15)	18.1022(14)	18.1543(13)	18.1274(14)
(Å)	*b*	20.4592(13)	20.4068(16)	20.4498(13)	20.4188(10)	20.4561(14)
	c	7.5218(4)	7.4959(5)	7.5082(4)	7.5102(4)	7.5147(5)
cell volume (Å^3)	*V*	2787.13(44)	2780.89(49)	2779.44(42)	2783.95(21)	2786.58(46)
Si(1)	*x*	0.1961(5)	0.1985(4)	0.20048(32)	0.2004(4)	0.19557(34)
16a	*y*	0.42821(30)	0.42581(33)	0.42849(24)	0.42823(27)	0.42793(26)
	z	0.5457(7)	0.5358(7)	0.5391(5)	0.5400(6)	0.5424(6)
	Occu.	0.99	0.99	0.99	0.99	0.99
	$U_{iso}{}^{b}$	0.0223(22)	0.0059(15)	0.0126(13)	0.0063(24)	0.0018(18)
Si(2)	*x*	0.19389(32)	0.1942(4)	0.19257(24)	0.19293(27)	0.19563(27)
16a	*y*	0.19053(24)	0.19022(29)	0.19087(19)	0.19090(21)	0.18987(21)
	z	0.5468(7)	0.5422(8)	0.5430(6)	0.5460(7)	0.5457(7)
Si(3)	*x*	0.08828(32)	0.0802(5)	0.08833(24)	0.08534(35)	0.08612(34)
8b	*y*	0.3808(6)	0.3817(6)	0.3810(4)	0.3819(6)	0.3792(4)
	z	0.25	0.25	0.25	0.25	0.25
Si(4)	*x*	0.0868(4)	0.0755(6)	0.08862(24)	0.0846(4)	0.0859(4)
8b	*y*	0.2217(6)	0.2213(6)	0.2230(4)	0.2219(6)	0.2204(5)
	z	0.25	0.25	0.25	0.25	0.25
O(1)	*x*	0.1225(6)	0.1248(5)	0.1255(4)	0.1268(5)	0.1219(5)
16a	*y*	0.4137(6)	0.4052(7)	0.4134(5)	0.4089(6)	0.4090(6)
	z	0.4300(8)	0.4282(11)	0.4276(7)	0.4286(7)	0.4321(7)
	Occu.	1	1	1	1	1
	U_{iso}	0.0417(37)	0.0145(34)	0.0451(34)	0.0247(36)	0.0213(27)
O(2)	*x*	0.1193(5)	0.1193(5)	0.1163(4)	0.1172(4)	0.1206(4)
16a	*y*	0.1867(6)	0.1976(7)	0.1861(5)	0.1892(6)	0.1876(5)
	z	0.4287(8)	0.4281(11)	0.4301(6)	0.4310(7)	0.4297(8)
O(3)	*x*	0.2618(6)	0.2703(6)	0.2689(4)	0.2693(5)	0.2635(5)
16a	*y*	0.3768(4)	0.3822(5)	0.37980(31)	0.37980(35)	0.37905(35)
	z	0.4981(21)	0.4841(23)	0.4926(17)	0.4952(22)	0.4952(18)
O(4)	*x*	0.0967(11)	0.0757(11)	0.1011(9)	0.0888(10)	0.0933(9)
8b	*y*	0.3011(6)	0.3014(6)	0.3020(4)	0.3018(6)	0.2998(4)
	z	0.25	0.25	0.25	0.25	0.25
O(5)	*x*	0.1629(7)	0.1685(10)	0.1640(7)	0.1616(7)	0.1662(7)
8b	*y*	0.1975(10)	0.8004(11)	0.1936(8)	0.1929(9)	0.1986(8)
	z	0.75	0.75	0.75	0.75	0.75
O(6)	*x*	0.1667(10)	0.1828(12)	0.1782(8)	0.1767(10)	0.1690(8)

8b	*y*	0.4230(9)	0.5831(10)	0.4232(8)	0.4219(9)	0.4219(7)
	z	0.75	0.75	0.75	0.75	0.75
O(7)	*x*	0.2306(10)	0.2283(10)	0.2371(7)	0.2367(8)	0.2298(8)
8b	*y*	0.5	0.5	0.5	0.5	0.5
	z	0.5	0.5	0.5	0.5	0.5
O(8)	*x*	0.25	0.25	0.25	0.25	0.25
8b	*y*	0.25	0.25	0.25	0.25	0.25
	z	0.5	0.5	0.5	0.5	0.5
O(9)	*x*	0	0	0	0	0
4c	*y*	0.3992(12)	0.4184(11)	0.3982(10)	0.4081(11)	0.4036(9)
	z	0.25	0.25	0.25	0.25	0.25
O(10)	*x*	0	0	0	0	0
4c	*y*	0.1992(12)	0.1776(11)	0.2069(9)	0.1944(11)	0.1951(10)
	z	0.25	0.25	0.25	0.25	0.25
M1	*x*	0	0	0	0	0.5
4c	*y*	0.5	0.5803(5)	0.5	0.5	0
	z	0	0	0	0	0
	Occu.	0.60	0.85	0.290(8)	0.186(7)	0.295(4)
	U_{iso}	0.171(14)	0.2267(57)	0.109(8)	0.126(11)	0.052(4)
M2	*x*		0	0	0	
4c	*y*		0.8504(6)	0.0052(15)	-0.0107(17)	
	z		0.25	0.75	0.75	
	Occu.		0.644(9)	0.330(13)	0.295(11)	
M2	*x*	0.4554(32)				0.5
8b	*y*	0.4040(24)				0.3817(9)
	z	0.25				0.0883(23)
	Occu.	0.55				0.127(2)
M3	*x*		-0.0527(29)	0.0842(18)	0.0769(19)	
8b	*y*		0.7333(14)	0.3960(21)	0.3977(20)	
	z		0.25	0.75	0.75	
	Occu.		0.1	0.180(5)	0.169(5)	
M3	*x*					0.4616(17)
16a	*y*					0.5567(14)
	z					-0.6306(34)
	Occu.					0.050(1)
M4	*x*					0.5
4c	*y*					0.105(8)
	z					0.25
	Occu.					0.030(3)
OW1	*x*	0			0	
4c	*y*	0.410(4)			0.3051(26)	
	z	0.75			0.75	
	Occu.	1			0.73(4)	
OW1	*x*		0.1545(19)	0.041(4)		0.4436(15)
8b	*y*		-0.0247(28)	0.2665(25)		0.2643(9)
	z		0.25	0.75		0.25
	Occu.		0.8	0.536(24)		1
OW1	*x*					

16a	*y*					
	z					
	Occu.					
OW2 *4c*	*x*	0		0	0	
	y	0.189(5)		0.1611(25)	0.1740(26)	
	z	0.75		0.75	0.75	
	Occu.	1		1	1	
OW2 *16a*	*x*		0.0736(6)			0.4145(10)
	y		0.9634(12)			0.5194(12)
	z		0.5484(34)			0.8346(27)
	Occu.		1			0.801(17)
OW3 *4c*	*x*		0			
	y		0.5			
	z		0			
	Occu.		0.002			
OW3 *16a*	*x*	-0.3960(15)		0.1076(21)	0.1135(25)	
	y	0.4905(19)		-0.0293(18)	-0.0304(22)	
	z	-0.188(7)		0.143(4)	0.177(8)	
	Occu.	0.755(1)		0.633(16)	0.5	
OW4 *4c*	*x*					
	y					
	z					
	Occu.					
OW4 *8b*	*x*	0		0	0	
	y	0.697(11)		0.0900(16)	0.0798(18)	
	z	0.370(25)		0.037(4)	-0.006(6)	
	Occu.	0.160(12)		1	1	

[a]Esd's are in parentheses. M denotes extra-framework cation sites; M is Na^+, Cs^+, Sr^{2+}, Cd^{2+}, and Pb^{2+} sites in the Na-MOR, Cs-MOR, Sr-MOR, Cd-MOR, and Pb-MOR model, respectively.

[b]Isotropic displacement factors (U_{iso}) were refined by grouping the framework tetrahedral atoms, the framework oxygen atoms, the non-framework contents, respectively.

Table S2. Selected interatomic distances (Å) and angles (˚) for Na-, Cs-, Sr-, Cd-, and Pb-MOR under ambient condition.[a]

	Na-MOR	Cs-MOR	Sr-MOR	Cd-MOR	Pb-MOR
Si(1) - O(1)	1.620(3)	1.619(4)	1.625(2)	1.626(2)	1.619(3)
Si(1) - O(3)	1.628(3)	1.628(4)	1.627(2)	1.630(3)	1.626(3)
Si(1) - O(6)	1.630(3)	1.640(4)	1.638(2)	1.640(2)	1.638(3)
Si(1) - O(7)	1.633(2)	1.631(3)	1.633(2)	1.634(2)	1.631(2)
mean[b]	1.628(1)	1.630(1)	1.631(1)	1.633(1)	1.629(1)
Si(2) - O(2)	1.619(3)	1.616(4)	1.623(2)	1.624(3)	1.617(3)
Si(2) - O(3)	1.630(3)	1.623(4)	1.627(2)	1.628(3)	1.622(3)
Si(2) - O(5)	1.635(3)	1.638(4)	1.639(2)	1.635(3)	1.635(3)
Si(2) - O(8)	1.624(3)	1.617(4)	1.627(2)	1.628(2)	1.613(3)
mean[b]	1.627(1)	1.624(1)	1.629(1)	1.629(1)	1.622(1)
Si(3) - O(1) x2	1.634(2)	1.634(4)	1.633(2)	1.634(2)	1.632(2)
Si(3) - O(4)	1.637(3)	1.641(4)	1.633(2)	1.636(3)	1.629(3)
Si(3) - O(9)	1.643(3)	1.640(4)	1.637(2)	1.639(3)	1.639(3)
mean[b]	1.637(1)	1.637(1)	1.634(1)	1.636(1)	1.633(1)
Si(4) - O(2) x2	1.633(2)	1.628(4)	1.627(2)	1.626(2)	1.634(2)
Si(4) - O(4)	1.634(3)	1.634(4)	1.632(2)	1.633(3)	1.630(3)
Si(4) - O(10)	1.639(3)	1.636(4)	1.638(2)	1.636(3)	1.641(3)
mean[b]	1.635(1)	1.632(1)	1.631(1)	1.630(1)	1.635(1)
Si(1) - O(1) - Si(3)	146.4(8)	153.4(9)	147.3(6)	152.0(7)	147.9(7)
Si(2) - O(2) - Si(4)	137.0(7)	151.0(10)	132.0(5)	138.1(6)	139.3(7)
Si(1) - O(3) - Si(2)	162.1(9)	147.7(10)	154.8(7)	154.3(7)	157.2(7)
Si(3) - O(4) - Si(4)	168.3(13)	177.3(13)	163.9(10)	175.2(11)	170.6(10)
Si(2) - O(5) - Si(2)	138.5(11)	144.0(13)	143.0(9)	139.1(10)	139.7(10)
Si(1) - O(6) - Si(1)	141.0(11)	156.2(14)	150.5(10)	148.2(11)	144.6(10)
Si(1) - O(7) - Si(1)	135.0(11)	141.2(13)	132.0(8)	132.5(9)	135.3(10)
Si(2) - O(8) - Si(2)	180	180	180	180	180
Si(3) - O(9) - Si(3)	153.5(16)		155.2(13)	141.9(13)	144.5(13)
Si(4) - O(10) - Si(4)	147.3(16)	114.0(14)	156.8(13)	139.8(14)	143.2(13)
M1 - O(1)	2.884(11)	3.325(11)	2.931(9)	3.008(11)	2.935(11)
M1 - O(6)	3.890(17)	3.324(22)			3.933(15)
M1 - O(9)	2.792(19)	3.303(22)	2.803(15)	2.655(15)	2.723(13)
		3.7481(3)			
M2 - O(2)	3.334(34)	3.389(11)			2.864(13)
M2 - O(5)	2.98(5)	3.229(19)			3.639(15)
M2 - O(10)		3.791(4)		3.75(4)	2.988(19)
M3 - O(1)		3.940(34)	2.558(12)	2.588(15)	
M3 - O(2)		3.046(23)			3.099(30)
					3.801(24)
M3 - O(3)			3.876(28)		
M3 - O(4)		3.837(10)			
M3 - O(5)		2.51(4)			
M3 - O(6)		3.87(5)	1.791(30)	1.88(4)	
M3 - O(7)			3.964(31)		
M3 - O(10)					3.051(28)
M4 - O(1)					3.267(17)
M4 - O(6)					3.112(34)
M4 - O(9)					3.762(8)
OW1 - O(1)	3.274(10)			3.951(31)	
OW1 - O(2)			3.213(32)	3.983(34)	2.848(15)
OW1 - O(3)		2.926(35)			
		3.78(5)			
OW1 - O(4)			3.975(24)		

OW1 - O(5)		3.58(6)	2.68(5)	3.72(4)	2.131(26)
OW1 - O(6)	3.030(18)	3.69(6)			
OW1 - O(7)		2.882(34)			
OW1 - O(8)					3.992(24)
OW1 - O(9)	3.768(6)				
	3.89(8)				
OW1 - O(10)					3.981(9)
OW2 - O(2)	3.243(9)	3.394(27)	3.235(10)	3.219(9)	3.921(26)
OW2 - O(3)		3.322(20)			3.641(24)
OW2 - O(5)	2.956(14)		3.042(16)	2.958(14)	3.996(29)
OW2 - O(7)		3.696(21)			3.594(21)
OW2 - O(10)	3.767(6)	3.516(26)	3.869(13)	3.778(6)	
OW3 - O(1)		3.030(14)			
OW3 - O(2)	3.74(4)		3.59(4)	3.73(5)	
OW3 - O(3)	3.65(4)		3.08(4)	3.09(5)	
	3.903(34)		3.918(32)	3.69(5)	
			3.95(4)	3.98(4)	
OW3 - O(5)			3.60(4)	3.47(5)	
OW3 - O(7)	3.318(34)		3.07(4)	3.70(5)	
	3.81(4)		3.931(33)		
OW3 - O(9)		2.506(14)			
OW4 - O(1)	3.50(18)				
OW4 - O(2)	3.56(15)		2.892(21)	3.136(29)	
OW4 - O(4)	3.35(16)				
OW4 - O(5)	3.77(14)				
OW4 - O(9)	3.47(22)				
OW4 - O(10)	3.56(18)		2.876(28)	3.03(4)	
OW1 - M1	2.63(6)				
OW1 - M2		3.79(6)			2.878(23)
OW1 - M3			2.76(5)	2.35(5)	
			3.49(6)		
OW1 - M4					3.42(16)
OW2 - M2	2.06(9)	3.480(27)	3.19(6)		2.614(22)
					3.150(22)
					3.739(28)
OW2 - M3			2.16(4)		1.80(4)
					2.346(34)
					2.502(25)
					2.745(30)
					3.133(30)
					3.251(27)
					3.68(4)
OW3 - M1		2.489(7)			
OW3 - M2	2.46(6)		2.16(4)	2.29(5)	
	3.48(6)		3.605(32)		
OW4 - M2			2.519(35)	2.38(4)	
			2.765(28)	2.60(4)	
	Na^+	Cs^+	Sr^{2+}	Cd^{2+}	Pb^{2+}
Bond Valence Sum (BVS)	0.68	0.83	2.13	1.14	1.17
Deviation from assumed valence state in valence units	0.32	0.17	0.13	0.86	0.83

[a]Esd's are in parentheses.

[b]Standard deviations computed using $\sigma = 1/n[\sum \sigma^2]^{1/2}$.
